# A Murine Inhalation Model to Characterize Pulmonary Exposure to Dry *Aspergillus fumigatus* Conidia

**DOI:** 10.1371/journal.pone.0109855

**Published:** 2014-10-23

**Authors:** Amanda D. Buskirk, Brett J. Green, Angela R. Lemons, Ajay P. Nayak, W. Travis Goldsmith, Michael L. Kashon, Stacey E. Anderson, Justin M. Hettick, Steven P. Templeton, Dori R. Germolec, Donald H. Beezhold

**Affiliations:** 1 Allergy and Clinical Immunology Branch, Health Effects Laboratory Division, National Institute for Occupational Safety and Health, Centers for Disease Control and Prevention, Morgantown, West Virginia, United States of America; 2 Pathology and Physiology Research Branch, Health Effects Laboratory Division, National Institute for Occupational Safety and Health, Centers for Disease Control and Prevention, Morgantown, West Virginia, United States of America; 3 Biostatistics and Epidemiology Branch, Health Effects Laboratory Division, National Institute for Occupational Safety and Health, Centers for Disease Control and Prevention, Morgantown, West Virginia, United States of America; 4 Toxicology Branch, National Toxicology Program Division, National Institute of Environmental Health Sciences, Research Triangle Park, North Carolina, United States of America; David Geffen School of Medicine at University of California Los Angeles, United States of America

## Abstract

Most murine models of fungal exposure are based on the delivery of uncharacterized extracts or liquid conidia suspensions using aspiration or intranasal approaches. Studies that model exposure to dry fungal aerosols using whole body inhalation have only recently been described. In this study, we aimed to characterize pulmonary immune responses following repeated inhalation of conidia utilizing an acoustical generator to deliver dry fungal aerosols to mice housed in a nose only exposure chamber. Immunocompetent female BALB/cJ mice were exposed to conidia derived from *Aspergillus fumigatus* wild-type (WT) or a melanin-deficient (*Δalb1*) strain. Conidia were aerosolized and delivered to mice at an estimated deposition dose of 1×10^5^ twice a week for 4 weeks (8 total). Histopathological and immunological endpoints were assessed 4, 24, 48, and 72 hours after the final exposure. Histopathological analysis showed that conidia derived from both strains induced lung inflammation, especially at 24 and 48 hour time points. Immunological endpoints evaluated in bronchoalveolar lavage fluid (BALF) and the mediastinal lymph nodes showed that exposure to WT conidia led to elevated numbers of macrophages, granulocytes, and lymphocytes. Importantly, CD8^+^ IL17^+^ (Tc17) cells were significantly higher in BALF and positively correlated with germination of *A. fumigatus* WT spores. Germination was associated with specific IgG to intracellular proteins while *Δalb1* spores elicited antibodies to cell wall hydrophobin. These data suggest that inhalation exposures may provide a more representative analysis of immune responses following exposures to environmentally and occupationally prevalent fungal contaminants.

## Introduction

Exposure to fungi derived from contaminated building materials are of growing concern in the general population [1]–[3]. Fungal exposures have been associated with multiple adverse health outcomes including invasive disease, allergic sensitization, hypersensitivity pneumonitis, and asthma [1]. Consensus documents published by the Institute of Medicine and the World Health Organization have identified associations between living in damp indoor environments containing mold and health effects, particularly asthma [4], [5]. Although sufficient evidence of associations exist, the fungal-specific factors and immunological mechanisms that lead to the induction of these allergic diseases require further characterization [6].

To date, numerous animal models of fungal exposure have been developed to investigate the immunological responses that follow fungal challenge [1], [7]–[9]. Although these studies have provided new insight, the test articles are often uncharacterized extracts, individual antigens or liquid spore suspensions that do not resemble typical human exposures. Many of these studies are based on a single exposure with few studies using repeated exposures [1], [10], [11], and even fewer studies utilizing inhalation exposures [12], [13].

To address the limitations associated with previous inhalation studies [12], [13], we developed a nose-only, acoustical generator exposure system (AGS) that allows for real-time analysis of particle size, deposition estimations, and manipulation of exposure concentrations. We used an immunocompetent murine model of repeated inhalation exposures with dry conidia to more closely model the burden of fungi encountered in the environment. *Aspergillus fumigatus* was chosen as the model organism to directly compare to previous exposure models, as well as previous studies conducted in our laboratory [14], [15]. Using this new system, we characterized the pulmonary immune responses to repeated inhalation of *A. fumigatus* conidia. Continuous monitoring of the real-time particle mass concentration in the animal's breathing zone allowed us to calculate estimates for the number of conidia that were deposited in the upper and lower respiratory tract. We additionally explored the response to *A. fumigatus* wild-type (WT) as well as a melanin-deficient (*Δalb1*) strain to examine the role of melanin and conidia germination on pulmonary immune responses following repeated dry fungal exposures in naïve mice.

## Materials and Methods

### Fungal culture

*A. fumigatus* strains B-5233/ATCC 13073 (wild-type (WT) parent strain) and *Δalb1* were received as a gift from Dr. June Kwon-Chung (NIAID, Bethesda, MD) [16]. Fungal cultures were grown for 14 days at room temperature (RT) on malt extract agar (MEA) as previously described [14]. For acoustical generation, a modified method was used to grow conidia [17]. In brief, 10 mL of sterilized, distilled, deionized water was added to one *A. fumigatus* MEA plate and conidia were suspended by disruption with a sterile inoculating loop. The fungal suspension (10 mL) was then used to inoculate 200 mL of dry brown rice (Mahatma brown rice, Allentown, PA) that was autoclaved (30 min, 121°C). The rice was completely submerged by the addition of 100 mL sterile water and approximately 10–12 g of wet rice was added to sterile 100 mm petri dishes. The plates were wrapped with parafilm and incubated at room temperature for 10–14 days with shaking once daily to prevent rice aggregates and ensure homogenous growth. Additional MEA plates were inoculated with the original suspension to ensure cultures were homogenous.

### Animals

Female BALB/cJ mice, aged 5–7 weeks (Jackson Laboratory, Bar Harbor, ME), were acclimated for approximately one week prior to exposures. The mice were housed in HEPA filtered, ventilated polycarbonate cages in groups of 5 on autoclaved hardwood chip bedding. Mice were provided with NIH-31 modified 6% irradiated rodent chow (Harlan Teklad) and tap water *ad libitum*. Sentinel mice were free of viral and bacterial pathogens. The National Institute for Occupational Safety and Health (NIOSH) animal facility is an environmentally controlled barrier facility that is fully accredited by the Association for the Assessment and Accreditation of Laboratory Animal Care International. All animal procedures were performed under a NIOSH Animal Care and Use Committee approved protocol 12-BG-M-003.

### Dry fungal aerosol exposure system

**Figure 1:**
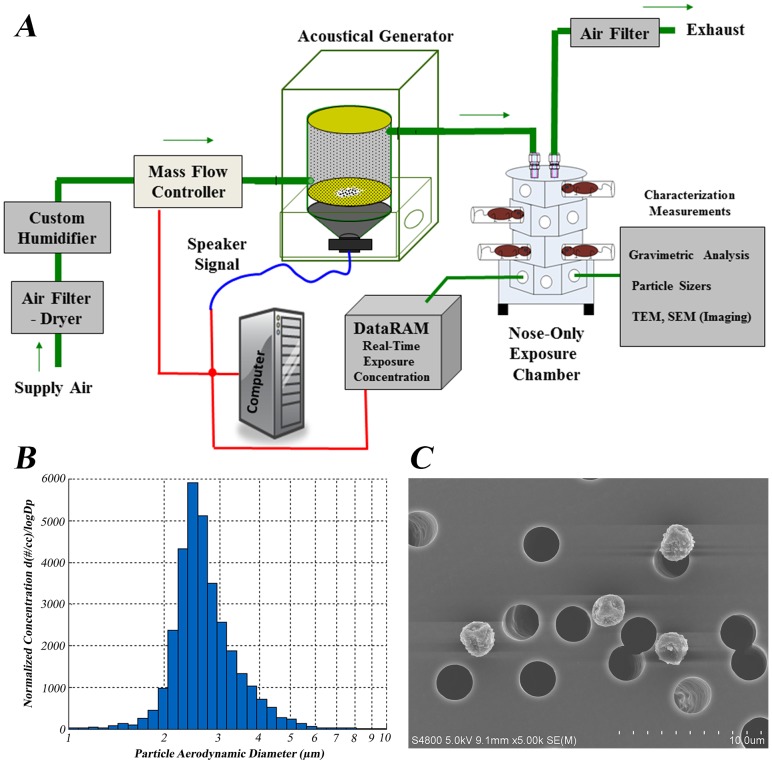
Preliminary acoustical generator exposure chamber experiments. A) Illustration showing the acoustical generator inhalation exposure system. Supply air is HEPA filtered and directed into the acoustical generator. The acoustical generator is then sent a signal to vibrate at a designated frequency resulting in the formation of fungal aerosols from inoculated rice grains. The fungal aerosol is then directed into a multi-animal, nose-only chamber. After passing through the animal's breathing zone the air is filtered before being sent into the exhaust system. A real-time particle counter attached to the computer calculates the concentration of fungal particles being deposited into the airways, and the DataRAM reports that number to the computer, which can be altered during the exposure to obtain the desired deposition concentration. B) The *Aspergillus fumigatus* aerosol particle size distribution produced by the acoustical generator. The size of single or aggregate *A. fumigatus* conidia is 2–5 µm. C) Field emission scanning electron microscopy image of *A. fumigatus* fungal conidia deposited on a polycarbonate filter collected from one of the sampling ports of the nose-only exposure chamber.

The nose-only inhalation AGS (Figure 1A) used conditioned air from a water seal compressor that passed through a dryer, a charcoal filter, and a high efficiency particulate air (HEPA) filter. The flow was regulated at 6 L/min by a mass flow controller (GFC37, Aalborg, Orangeburg, NY). The conditioned air flowed through a modified PITT-3 AGS consisting of a speaker covered with a rubber membrane, on which rice cultures were placed and allowed to settle 12–24 hours prior to exposure. Acoustical energy was then used to vibrate the conidia-laden rice resulting in the detachment, de-aggregation, and aerosolization of the conidia within the generator. The air-conidia mixture then passed into a modified nose-only exposure chamber (Intox Products, LLC, Moriarty, NM). The conidia entered the top of the chamber into a column where animals were positioned in separate pods that projected out radially from the column. The conidia entered the pods directly in front of the nose of the animals and exhaled air exited via radial ports around the nose of the mouse into a second column where the air was HEPA filtered and exhausted. Of 24 pods, 2 pods were reserved as sample ports; one was used to gravimetrically measure the conidial mass concentration and the second used a light scattering device (DataRAM4, ThermoElectron Co., Franklin, MA) to provide a real-time estimate of the mass concentration of the conidia in the exposure chamber. The DataRAM also recorded the chamber temperature and humidity. During test runs, the gravimetric sample port was used to collect samples for electron microscopy and to size the particles with an aerodynamic particle sizer (APS, TSI Inc., Shoreview, MN; Figure 1B and 1C).

A mouse lung deposition model, based on deposition measurements from Raabe *et al.* [18], was developed to allow for estimation of the number of conidia deposited within the murine lung. The conidia frequency in each size bin of APS measurements were cross-referenced to the corresponding interpolated mouse lung deposition fraction for that particle size. A scale factor was established and applied to the DataRAM signal to estimate the real-time number of conidia deposited based on the DataRAM's mass concentration measurements and a mouse minute volume of 25 mL. The integrated value of deposition was calculated throughout the exposures using custom software and the system automatically turned off when the desired conidial deposition was reached (∼100 min). Animals were acclimated to the exposure chamber by placing them in the nose-only housing units for increasing time intervals (up to 2 hours), over the course of a one week period prior to the first exposure. The acclimation was to reduce potential stress or other adverse conditions resulting from prolonged restraint required for the fungal exposures.

For exposures, fifteen mice per time point were exposed to *A. fumigatus* WT conidia, *Δalb1* conidia, or HEPA filtered air only. Mice were placed individually in the exposure units attached to the acoustical generator for approximately 2 hours, while the generator was automatically turned off when real-time particle dose estimates reached 1×10^5^ conidia. Mice were exposed twice per week (Thursday and Monday or Friday and Tuesday) for 4 weeks, and sacrificed at 4, 24, 48, or 72 hours post-final exposure using an intraperitoneal injection of 100 uL (100 mg/kg body weight) of sodium pentobarbital (Sleepaway, Fort Dodge Animal Health, Fort Dodge, IA).

### Modified local lymph node assay

Mice were exposed to 5×10^3^, 1×10^4^, 1×10^5^, 1×10^6^ WT conidia or HEPA filtered air only via the AGS exposure chamber once daily for three days, and then rested for two days. To serve as a positive control for exposures, a group of mice were exposed to 200 µg of hyphal extract via pharyngeal aspiration using the same exposure schedule. On the sixth day, mice were injected intravenously via the lateral tail vein with 20 µCi 3H-thymidine (Dupont NEN, Waltham, MA; specific activity 2 Ci/mmol). Five hours following 3H-thymidine injection, animals were euthanized via CO_2_ inhalation. The mediastinal lymph nodes (MLNs), located at the bifurcation of the bronchi in the lung, were removed for analysis. MLNs were homogenized between frosted microscope slides, cell suspensions were prepared, and samples were incubated with 5% trichloroacetic acid overnight at 4°C. Samples were then mixed with scintillation fluid and counted using a Tri-Carb 2500TR liquid scintillation counter. Stimulation indices (SIs) were calculated by dividing the mean disintegrations per minute (DPM) by the mean DPM obtained from the control chamber exposure mice. Pre- and post-weight data was additionally recorded to assess potential toxicity. Concentrations of conidia that induced greater than 10% weight loss were not considered for the repeated exposures, despite the SI value.

### Histology

For each experiment, three mice per time point were selected for histology analysis. Lungs were dissected and fixed as previously described [14]. Tissues were sectioned and stained with hematoxylin and eosin (H&E), Periodic Acid Shift (PAS), and Grocott's Methenamine Silver (GMS) staining.

Conidia were identified using GMS stain and quantified by counting the number visualized in 100 random fields of view (400× magnification), covering both lungs fields. Conidia >2× the size of resting conidia were classified as swollen and the emergence of germ tubes from conidia were classified as germinated. Data were presented as the total conidial counts per exposure or the percentage of total conidia that were germinating (swollen+germinated).

### Collection of bronchoalveolar lavage fluid, mediastinal lymph nodes, and serum

Bronchoalveolar lavage fluid (BALF) and serum was collected as previously described [14]. Serum was collected from mice 7 days following final exposure to WT and *Δalb1* conidia. Following collection of BALF, the MLNs were dissected, cleared of adipose tissue and homogenized by grinding the nodes between frosted microscope slides. Cells isolated from the BALF and MLN were then prepared for flow cytometry analysis.

### Differential cell staining

Cellularity was determined by flow cytometry using reagents obtained from BD Biosciences (San Jose, CA) unless otherwise specified. The resuspended BALF and MLN cells were divided in two for differential and intracellular cytokine staining. One half of the BALF cells were stained for 30 minutes using the following antibodies at 1∶100 rat anti-mouse Ly-6G, rat anti-mouse Siglec-F, pan-leukocyte rat anti-mouse CD45, and Armenian hamster anti-mouse CD11c (eBioscience Inc. San Diego, CA). MLN cells were also stained to identify B cell populations using B220/CD45R antibodies. After staining, cells were washed and fixed with BD Cytofix. Cells were then washed and resuspended in FACS buffer.

Cell populations were evaluated on a BD LSRII (BD Biosciences, San Jose, CA). Neutrophils were defined as CD45^hi^Ly-6G^hi^, eosinophils as Ly-6G^low^SiglecF^hi^CD11c^low^, and alveolar macrophages as Ly-6G^low^SiglecF^hi^CD11c^hi^ as previously reported [14], [19]. Total cell numbers were quantified using acridine orange nuclear staining and an automated cell counter (Cellometer AutoX4, Nexcelom Bioscience, Lawrence, MA). Total numbers of each cell population were obtained by multiplying the frequency of specific population by the total number of BALF cells recovered for each animal.

### Intracellular cytokine staining

BALF and MLN T-cells were quantified using rat anti-mouse CD8 and CD4 antibodies. T-cell cytokine production was determined by fluorescent intracellular cytokine staining (ICS) as previously described [20]. Briefly, the BALF and MLN suspensions were washed and incubated in Leukocyte Activation Cocktail with GolgiPlug at 37°C in 5% CO_2_ for 4 hours. Following incubation, the BALF cells were washed and stained for flow cytometry analysis using rat anti-mouse CD4, rat anti-mouse CD8, rat anti-mouse B220/CD45R, and rat anti-mouse CD25. MLNs were stained using Syrian hamster anti-mouse CD3e, rat anti-mouse CD4, and rat anti-mouse CD8 antibodies. Cells were then washed, resuspended in BD Cytofix/Cytoperm, and incubated for 15 minutes at 4°C. Cells were washed and resuspended in PermWash. Each BALF sample was stained 1∶100 with rat anti-mouse IFN-γ, rat anti-mouse IL-13, rat anti-mouse IL-10, and rat anti-mouse IL-17A (eBioscience, San Diego, CA). MLNs were stained with rat anti-mouse IL-13, (BioLegend, San Diego, CA), rat anti-mouse IL-10, rat anti-mouse IL-12, rat anti-mouse IL-22, rat anti-mouse IL-17A, and rat anti-mouse IFN-γ. Cell populations were analyzed on a BD LSRII, with lymphocytes gated on the basis of low forward and side scatter, then subsequently gated on CD4^+^ or CD8^+^ populations to determine intracellular expression of cytokines.

### *Aspergillus fumigatus* protein extraction

Conidial protein extracts from WT and *Δalb1* conidia were obtained from 14 day old MEA plates. Conidia were harvested from the plates by adding 5 mL of sterile, distilled, deionized water, and agitating the surface of the agar with a sterile inoculating loop. Samples were frozen overnight at −80°C, and then lyophilized for 3–5 days to remove the suspension solution. Glass beads (0.5 mm) were added to each sample. Conidia were then subjected to 3×1 minute bead beating cycles using a mini-bead beater (BioSpec, Bartlesville, OK). Sodium bicarbonate buffer (pH 8.0, 2 ml), containing 0.5 mM EDTA and protease inhibitors (Complete Mini, Roche Diagnostics, Indianapolis, IN) was added and the samples were agitated by three one-minute bead-beating cycles, centrifuged, and the resulting supernatant was used for SDS-PAGE.

For Western blot and ELISA analyses, hyphal extracts were prepared by harvesting mycelial cultures by centrifugation at 4,100× *g* for 5 min. The hyphal pellet was collected and concentrated by lyophilization. Lyophilized hyphae were then macerated in a mortar and pestle containing liquid N_2_ and suspended in cold PBS, pH 7.4, containing Complete Mini Protease Inhibitor Cocktail (Roche Diagnostics, Indianapolis, IN). Proteins were extracted overnight at 4°C on a rocker. The extract was then centrifuged at 4,100× *g* for 5 min, and the supernatant fluid was collected, aliquoted, and stored at −80°C for further analysis.

For two-dimensional Western blot analysis, mycelial extracts were prepared based on previously published methods [21]. Briefly, hyphae were ground in liquid nitrogen using a mortar and pestle and resuspended in 10% (w/v) trichloroacetic acid (TCA). After centrifugation at 20,200× *g* for 10 min at 4°C, samples were washed twice in ice cold acetone. The pellets were then resuspended in IEF buffer (10 mM Tris, 8 M urea, 2 M thiourea, 4% (w/v) CHAPS, 1% (v/v) Triton X-100, 65 mM dithiothreitol (DTT) and 0.8% (w/v) IPG buffer), centrifuged at 14,000× g for 5 min at 4°C, and the resulting supernatant was used for analysis. Protein concentrations in all fungal extract preparations were determined using a NanoDrop ND-1000 spectrophotometer (NanoDrop Technologies, Wilmington, DE).

### SDS PAGE and Western blot analysis

*A. fumigatus* WT and *Δalb1* conidial and hyphal extracts (10 µg) were prepared as described above and mixed with Laemmli sample buffer (Bio-Rad, Hercules, CA) containing β-mercaptoethanol and heated at 95°C for 5 minutes. Samples were loaded in individual lanes (10 µg/well) and resolved using a 12% polyacrylamide gel with a 4% stacking gel ran at 100 V for 90 minutes. Proteins were transferred to a 0.2 µm nitrocellulose membrane at 16 V overnight at 4°C. The membrane was blocked for 1 hour with 5% non-fat dry milk powder (SM) in Tris-buffered saline containing 0.05% Tween-20 (TBST). Pooled polyclonal sera from mice 7 days post exposure was diluted to 1∶200 in SM-TBST and incubated with the membrane at room temperature (RT) for 1 hour with gentle agitation. The blot was then washed 3 times with TBST and incubated with alkaline phosphatase (AP)-conjugated goat anti-mouse IgG heavy and light chain (Promega, Madison, WI) diluted 1∶5000 in SM-TBST for 1 hour (RT) with gentle agitation. The blot was then washed 3 times with TBST and developed using the AP substrate, 1-Step NBT/BCIP Solution (Thermo Scientific, Rockford, IL).

### Two-dimensional Western blot analysis

*A. fumigatus* B5233 hyphal proteins (500 µg) prepared in IEF buffer as described above were suspended in 130 µl sample solubilization buffer (8 M urea, 2% (w/v) CHAPS, 0.5% (w/v) IPG Buffer, 20 mM DTT, trace bromophenol blue) containing 0.625 µl ampholytes (3–10, GE Healthcare, Uppsala Sweden). The protein was then loaded onto a 7 cm ReadyStrip IPG strip, pH 3–10 (GE Healthcare) and focused at 4000 V for 15 kVh and held at 500 V (Protean i12 IEF Cell, BioRad, Hercules, CA). IPG strips were equilibrated for 15 min at RT in equilibration buffer (6 M urea, 2% (w/v) SDS, 50 mM Tris/HCl, pH 8.8, 20% (v/v) glycerol) containing 130 mM DTT, followed by a second 15 min incubation in equilibration buffer containing 135 mM iodoacetamide. The equilibrated IPG strip was then placed on a 1.5 mm, 12% gel, electrophoresed at 100 V for 1.5 hr and transferred overnight at 15 V to a nitrocellulose membrane (0.2 µm, BioRad). Western blot analysis was performed as previously described.

### Proteomic characterization of murine IgG reactive *A. fumigatus* antigens

Murine IgG reactive *A. fumigatus*-specific protein bands were identified by Western blot and excised from imperial stained SDS-PAGE gels prepared in parallel. Excised spots were digested with porcine trypsin (Sigma Aldrich) and analyzed using a nanoACQUITY UPLC coupled to a SYNAPT quadrupole time-of-flight mass spectrometer (qTOFMS; Waters, Milford, MA) according to previously published methods [22], [23].

### Total IgE ELISA

Total IgE titers in pre-bleed sera and pooled polyclonal sera isolated from mice exposed to WT and *Δalb1* strains were determined using a total IgE kit, as per the manufacturer's instructions (Biolegend, San Diego, CA). Sera were prepared at 1∶10 and serially diluted and plates were read at 450 and 570 nm. Background absorbance values obtained from the 570 nm readings were subtracted from the 450 nm values. The standard curve was created from known concentrations of IgE standards, and unknown IgE sample concentrations were extrapolated from the curve.

### Specific IgG ELISA

Specific IgG concentrations in pooled polyclonal sera obtained 7 days post exposure to WT and *Δalb1* strains were determined using an indirect ELISA. Briefly, Corning high protein binding 96-well plates (Corning, NY) were coated with 3 µg/mL *A. fumigatus* conidial or hyphal extract in 0.1 M sodium carbonate, pH 9.6 overnight at 4°C. The wells were washed three times with PBS containing 0.05% Tween 20 (PBST) and blocked with 200 µL/well 3% non-fat dry milk powder in PBST (SM-PBST) for 1 hour at 37°C. Duplicate wells were incubated with mouse sera diluted 1∶200 in SM-PBST for 1 hour at 37°C. Wells were washed and incubated for 1 h at 37°C with AP-conjugated anti-mouse IgG heavy and light chain antibody (Promega) diluted 1∶5000 in SM-PBST. Wells were washed and developed with 0.5 mg/mL *p*-nitrophenyl phosphate in AP substrate. The optical density was determined spectrophotometrically at 405 nm using a SpectraMax M4 microplate reader (Molecular Devices, Sunnyvale, CA).

### Data analysis methods

Analysis of flow cytometric samples was performed with BD FACSDiva software (BD Biosciences, San Jose, CA). SigmaPlot (Systat Software, Inc., San Jose, CA) was used for graphs, and GraphPad Prism (GraphPad Software, La Jolla, CA) for statistical analysis of conidial clearance/germination and total IgE levels. The data were analyzed via two way analysis of variance (ANOVA) followed by Bonferroni post-test. Statistical analysis of cellularity was performed using SAS version 9.3 for Windows (SAS, Cary, NC). The data were log transformed prior to analysis and Proc Mix was used to run a two-way factorial analysis of variance (Fungal Exposure by Time). Pairwise comparisons were performed using Fishers Least Significant Difference test. Differences between experimental groups were considered significant with p-values ≤0.05.

## Results

### Characterization of the fungal acoustical generator system

A new inhalation exposure system was developed to enable characterization of pulmonary immune responses to dry particles [24]. Figure 1A depicts a schematic of the AGS adapted for use with fungal conidia. The AGS was capable of producing mass concentrations of up to 60 mg/m^3^ (count concentration 2.6×10^9^ conidia/m^3^; data not shown) and field emission scanning electron microscopy (FESEM) showed aerosols composed of individual conidia, and in some cases aggregate conidia (Figure 1B and 1C), with a size distribution within the aerodynamic diameter of individual conidia (Figure 1B). Rice grain fragments or fungal fragments (fractured conidia or hyphae) were not observed by FESEM. These findings demonstrated that conidia could be aerosolized in reproducible quantities and delivered to mice housed in the multi-animal nose only exposure chamber. Histopathological assessment of animals in a pilot study confirmed that fungal conidia were deposited within the upper respiratory tract and in the smaller airways (data not shown).

### Lymphoproliferation induced by repeated dry *A. fumigatus* exposures

The optimal dose for inhalation experiments was determined by a modified local lymph node assay following 3 repeated inhalation exposures to *A. fumigatus* WT spores (5×10^3^, 1×10^4^, 1×10^5^, or 1×10^6^ calculated airway deposition, CAD). Proliferation of lymphocytes as determined by the incorporation of radiolabeled tritiated thymidine in the MLNs was dose dependent and 1×10^6^ conidia (CAD) induced the highest stimulation index (∼100 SI units (fold change over vehicle control); Figure S1); however, mice exposed to this dose had greater than 10% weight loss so 1×10^5^ was selected for future experiments. This dose corresponds to the fungal burden that may be encountered in contaminated indoor and some occupational environments [1].

### Histopathology

**Figure 2:**
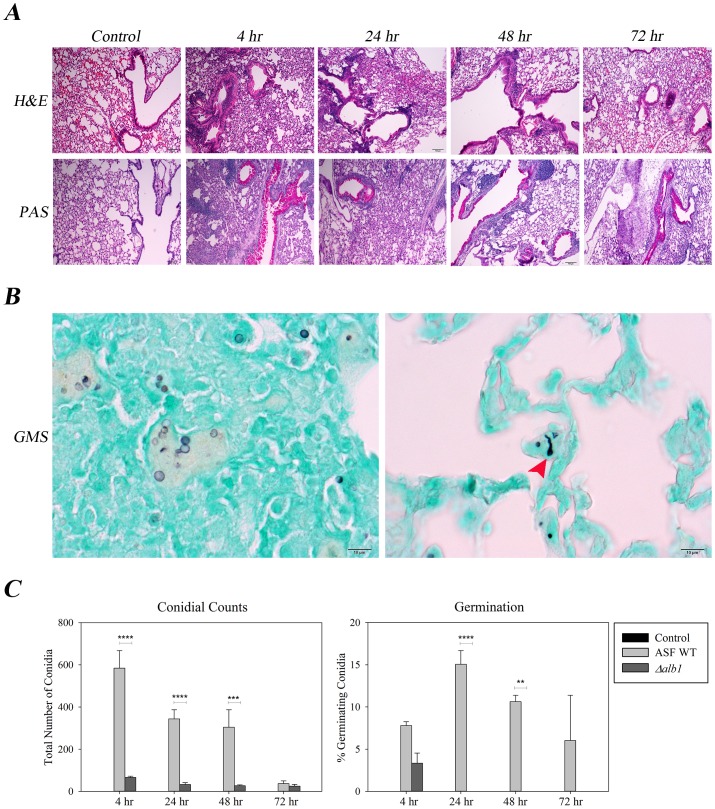
Histopathology of sections derived from *Aspergillus fumigatus* WT exposed mice. A) Representative histopathology sections from WT exposed mice sacrificed at the indicated time points. Top panel-H&E stained sections at 100× magnification, Bottom panel-PAS stained sections at 100× magnification. B) GMS stained sections at 400× magnification. Black arrow heads indicate swollen conidia (24 hr), while red arrow heads indicate conidia germination and emergence of hyphal tubes (48 hr). C) Quantification of conidia and germination (swollen conidia+germ tube formation) over time. Values were obtained by quantifying the number of conidia visualized by counting 100 random fields of view covering both lung fields at a magnification of 400×. Conidia were considered swollen when the size was >2× that of resting conidia. Data are presented as the average ± standard error of measure. ****P≤0.0001, ***P≤0.001, **P≤0.01.

Using 1×10^5^ spores, experiments were performed to characterize the pulmonary response to repeated fungal conidia exposures. After 8 exposures, H&E staining revealed inflammation predominantly affecting the bronchioles, characterized by leukocyte infiltration (Figure 2A) and giant cell formation (data not shown). Airway remodeling, increased mucus production and goblet cell hyperplasia were also observed (data not shown). The time course revealed that by 24 hours, mucus production had begun to resolve and the cellularity was largely composed of neutrophils and lymphocytes surrounding the bronchioles (Figure 2A). Granulomas were occasionally observed near the larger airways (data not shown). Compared to WT exposures, mice exposed to *Δalb1* conidia did not have as pronounced inflammation; however, airway remodeling, increased mucus production, and goblet cell hyperplasia were observed (Figure S2).

Within 4 hours of final exposure, GMS stained WT conidia could be microscopically observed in the lung interstitium. Some conidia were swollen and minimal germ tube formation was present at this time point (Figure 2B and C). Considerably fewer conidia (mean = 66.5) were resolved in the lungs of mice exposed to *Δalb1* conidia at 4 hours (Figure 2C). By 24 and 48 hours, germination of WT conidia was significantly increased compared to *Δalb1* conidia that had not germinated (Figure 2C). By 72 hours, most inflammatory changes observed in histopathology were resolving and few conidia were observed (Figure 2).

### BALF Cellularity

**Figure 3:**
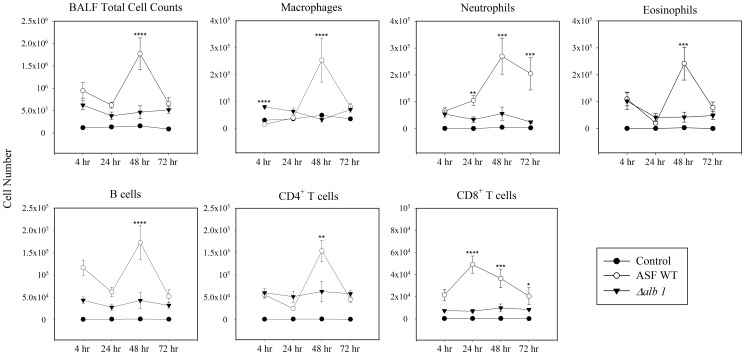
Total cell counts in the BALF. Following 8 dry conidial exposures, mice were sacrificed at the indicated time points to determine the kinetics of the cellular influx to the lung. Total cell numbers were obtained through acridine orange staining and quantified using an automated cell counter. Each cell population was quantified by multiplying the frequency of each by the total cell counts. Data are presented as the average ± standard error of measure. (Control n = 30 mice/time point, Exposed n = 7–10 mice/group/time point). ****P≤0.0001, ***P≤0.001, **P≤0.01, *P≤0.05.

Quantification of total BALF cell numbers showed a statistically significant difference in the cellular influx between the WT exposure group and controls. Mice exposed to WT conidia had significantly higher BALF cell counts (monocytic cells and eosinophils) at 48 hours with significantly elevated neutrophil counts persisting at 72 hours (Figure 3). Compared to WT conidia, BALF cellularity in mice exposed to *Δalb1* conidia was generally lower and peaked for most cell types by 4 hours (Figure 3).

**Figure 4:**
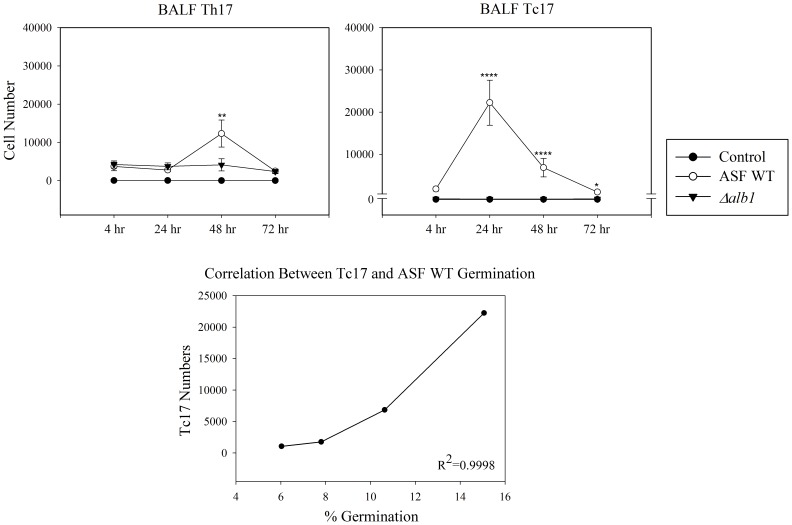
Intracellular cytokine flow cytometry analysis of the BALF. IL-17^+^ CD4^+^ and CD8^+^ T cells were quantified by multiplying the frequency of each individual cell population by the total cell counts. Mice were sacrificed at the indicated time points after the 8th exposure. Data are presented as the average ± standard error of measure. n = 7–10 mice/group per time point. ****P≤0.0001, **P≤0.01.

To further characterize the pulmonary immune responses, lymphocyte subpopulations were examined. Exposures to WT conidia resulted in peak CD8^+^ T cell counts at 24 hours followed by a gradual decline to 72 hours. A similar response was not observed with the *Δalb1* conidia. CD4^+^ T cell responses showed a sharp peak at 48 hours following WT exposures; however, the levels of this cell population at the other time points were similar to *Δalb1* exposed mice (Figure 3). Intracellular cytokine staining of the BALF cells revealed mice exposed to WT conidia recruited significantly more CD4^+^IL-17^+^ T cells (Th17) to the airways at 48 hours compared to the *Δalb1* exposure group (Figure 4). Interestingly, CD8^+^IL-17^+^ T cells (Tc17) reflected a majority of the overall CD8^+^ T cell response observed in WT exposed mice and this population peaked at the 24 hour time point (Figure 4). Additional lymphocyte phenotypes demonstrating a mixed Th1/Th2 response can be found in File S1, Figure S3.

### Mediastinal Lymph Node Cellularity

**Figure 5:**
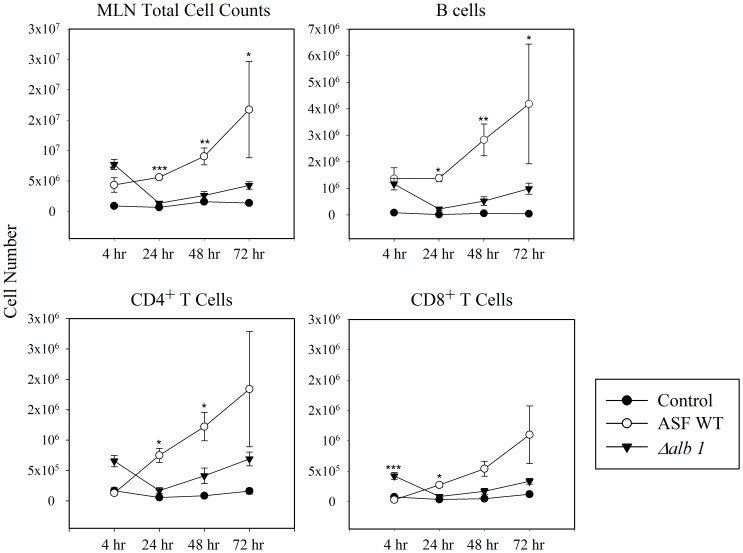
Total cell counts in the MLNs. Following 8 dry conidial exposures, mice were sacrificed at the indicated time points to determine the cellular influx kinetics to the lung-associated nodes. Total cell numbers were obtained through acridine orange staining and quantified using an automated cell counter. Each cell population was quantified by multiplying the frequency of each by the total cell counts. Data are presented as the average ± standard error of measure. (Control-n = 30 mice/time point, Exposed n = 7–10 mice/group/time point). ***P≤0.001, **P≤0.01, *P≤0.05.

In the draining mediastinal lymph nodes (MLN), the numbers of total MLN cells and B cells increased up to 72 hours in WT exposures; however, in the *Δalb1* exposure group, the highest MLN and B cell counts were observed at 4 hours (Figure 5). These counts were reduced at 24 hours, and began to increase slightly again by 72 hours (Figure 5).

WT exposures resulted in a steady increase in CD4^+^ T cells up to 72 hours, while in *Δalb1* exposure, CD4^+^ T cells were highest at 4 hours (Figure 5). Intracellular cytokine staining revealed that Tc17 responses also increased with each time interval and reached highest levels at 72 hours following the final exposure to WT conidia (data not shown). Additional lymphocytic phenotypes determined by intracellular cytokine staining can be found in Figure S4 and indicated a mixed Th1/Th2 response.

### *Aspergillus fumigatus*-specific antibody production

**Figure 6:**
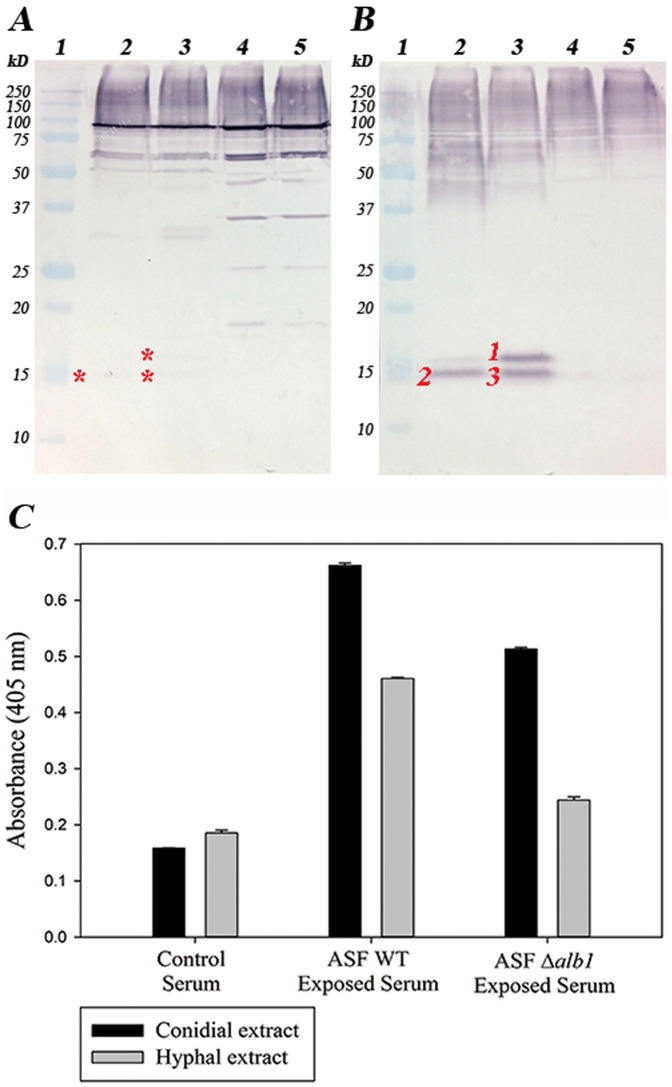
Specific IgG from *A. fumigatus* WT or *Δalb1* exposed mice. Western blot analysis of specific IgG in serum from mice exposed to acoustically aerosolized conidia from (A) WT or (B) *Δalb1* strains. Lanes 1-molecular weight markers, 2-ASF WT conidial extract, 3-*Δalb1* conidial extract, 4-ASF WT hyphal extract, 5-Δalb1 hyphal extract (10 µg protein/lane). Numbered bands were excised and identified using LC/MS analyses. The asterisks (*) denote weak binding of WT–exposed serum antibody to hydrophobin. C) ELISA analysis of IgG specific for *A. fumigatus* WT and *Δalb1* conidial proteins or hyphal extracts. Results are representative of the mean OD_405_ values for each mouse sera diluted 1∶200 ± the standard deviation of duplicate ELISA wells coated with 3 µg/mL protein.

ELISA analysis of *A. fumigatus*-specific antibody showed presence of IgG at 7 days (Figure 6C) but IgM was not detected in either group (data not shown). In Western blots, WT exposed sera reacted to a greater number of bands in hyphal extracts compared with conidial extracts from both WT and *Δalb1* strains (Figure 6A). Conversely, *Δalb1* exposed sera reacted to fewer proteins; however, immunostaining of conidial proteins showed prominent immunoreactive bands localized between 10–15 kDa in both WT (band 2) and *Δalb1* (band 1 and 3) conidia extracts (Figure 6B).

Proteomic analysis was performed on protein bands identified to bind sIgG. Prominent bands from WT and *Δalb1* hyphal extracts were identified at approximately 85 kDa, 65 kDa, and 52 kDa. These proteins were further resolved by two-dimensional Western blot (Figure S6, Table 1, Table 2). LC-MS/MS analysis of the hyphal proteins identified to bind WT sIgG showed that many of these spots were derived from intracellular proteins with a range of functions (Table 2). Protein spots 1–2 (∼85 kDa), 3 (∼65 kDa), 4 (∼60 kDa) and 6 (41 kDa) were identified as mitochondrial proteins involved in various biosynthetic and metabolic processes. Other proteins binding WT sIgG were identified as enzymes involved in metabolic processes in the cytosol, namely spot 5 (52 kDa) identified as proteasome regulatory particle subunit Rpt3 and spot 7 (37 kDa) identified as glyceraldehyde 3 phosphate dehydrogenase (GAPDH). Interestingly, the prominent immunoreactive conidial proteins recognized by the *Δalb1* conidia exposed sera (between 10–15 kDa) were identified in both WT (band 2) and *Δalb1* (bands 1 & 3) to be predominantly derived from hydrophobins (Table 1).

**Table 1:** Proteomic analysis of immunoreactive conidia protein bands identified in sIgG Western blot.

Protein bands	Protein Identification	Protein Accession Number	Theoretical molecular mass (kDa)/pI	Number of peptides	Sequence coverage (%)	Predicted signal peptide (positions)
1	a) Hydrophobin	P41746	16.11/5.17	26	66.04	Yes (1–18)
	b) Nucleoside diphosphate kinase	B0Y2U5	16.93/7.76	12	37.91	No
2–3	a) Hydrophobin	Q8TFV2	11.42/4.66	20	46.73	No
	b) Putative, uncharacterized protein	B0YDB8	12.85/5.73	2	24.56	No

**Table 2:** Proteomic analysis of immunoreactive hyphal protein bands identified in WT sIgG two-dimensional Western blot.

Protein spots	Protein Identification	Protein Accession Number	Theoretical molecular mass (kDa)/pI	Numberof peptides	Sequence coverage (%)
1	a) 5-methyltetrahydropteroyltriglutamate homocysteine S-methyltransferase	B0Y5S8	86.84/6.33	3	4.01
2	a) Putative, Mitochondrial aconitate hydratase	Q4WLN1	85.48/6.25	8	9.91
3	a) Putative, Mitochondrial dihydroxy acid dehydratase	B0XTT1	68.21/7.78	10	13.08
	b) Phosphoglucomutase PgmA	B0XXA2	60.46/6.30	14	16.58
	c) Putative, Pyruvate decarboxylase PdcA	B0XXN9	62.96/6.07	3	10.72
	d) Putative, GMC oxidoreductase	Q4WFN7	72.10/7.08	3	7.58
4	a) Putative, Antigenic mitochondrial protein HSP60	B0XRX3	61.91/5.35	35	37.99
5	a) Putative, Proteasome regulatory particle subunit Rpt3	Q4WY11	51.57/4.99	11	26.02
6	a) Putative, Acetyl CoA acetyltransferase	B0YA65	40.88/6.43	12	17.59
7	a) Glyceraldehyde 3 phosphate dehydrogenase (GAPDH)	B0Y207	36.29/7.27	28	33.14

Serum IgE was additionally quantified to further characterize the immune response following repeated *A. fumigatus* WT and *Δalb1* conidia exposures. Total IgE concentrations were significantly increased in each exposure group despite the presence of melanin (Figure S5). Similar to the Western blot data, IgE titers increased by approximately 1000 ng/mL (WT exposed) and 1500 ng/mL (*Δalb1*) between days 3 and 7. Overall, these data demonstrated that nose-only exposures to dry *A. fumigatus* conidia may result in the production of sIgG, as well as total IgE.

## Discussion

Animal models of fungal exposure have provided understanding of the pulmonary immune mechanisms that mediate fungal clearance and pathogenesis [9]–[11]. However, many of these studies use pharyngeal and intratracheal aspiration or intranasal methodologies to deliver uncharacterized extracts or conidia suspensions to animals [1], [9]–[11]. These variables may not represent exposure to fungal bioaerosols typically encountered in the environment. Recently, several animal studies have evaluated inhalation exposures to aerosolized fungal conidia that more closely reflect human exposures to indoor, outdoor, or occupational environments [12], [13], [25]. In this study, we describe a system that more closely represents typical inhalational exposures to fungal conidia and the pulmonary immune responses in immunocompetent mice following repeated (n = 8) exposures to a lung dose of 1×10^5^ dry *A. fumigatus* conidia. It is estimated that this lung burden would be equivalent to a worker breathing a constant conidia workplace air level concentration of 5.60×10^4^ spores/m^3^ (0.93 µg/m^3^) over a 45 year working career. This daily dose could be encountered in contaminated occupational environments [1].

An innovative AGS, based on a similar system developed at NIOSH [26] was used to produce homogenous *A. fumigatus* WT and *Δalb1* (melanin deficient) conidia aerosols that were delivered to mice housed in a multi-animal nose only exposure chamber. The AGS allows the controlled delivery of fungal conidia to mice, limits fungal bioaerosol deposition distal to the neck, and reduces supplementary exposures such as the ingestion of conidia associated with barbering. The inhalation of the dry aerosol also avoids alterations to the conidia as a result of preparation of fungal extracts or liquid conidia suspensions. The AGS uses mass concentration measurements to estimate particle deposition numbers so that exposure/deposition could be kept constant throughout the duration of the exposures. These variables could not be controlled in previously published murine inhalation models of *A. fumigatus* exposure [12],[13].

Previously, we published an aspiration exposure model of repeated exposures *to A. fumigatus* mutant strains [14]. Mice were exposed twice weekly for 4 weeks and harvested 72 hours after the final exposure. In that study, we observed moderate to severe inflammation resembling hypersensitivity pneumonitis, and while similar inflammatory changes were observed between mutant strains of *A. fumigatus*, histological differences in conidia clearance were readily apparent. In the present study, we extended these observations to an inhalation exposure and we examined 4 time points post exposure to characterize the dynamics of the immune response of mice. Histopathologic analysis revealed a severe inflammatory response within 4 hours of the final exposure. Influx of innate cells could be observed, as well as conidia transported from the airways into the surrounding lung interstitium. Mucus production by goblet cells, bronchiolar pneumonia, and tissue remodeling were also observed. Large numbers of neutrophils and eosinophils were observed surrounding the larger airways and giant cell formation and granulomas were apparent. Overall, the inflammation observed in the present study was not as severe compared to previous *A. fumigatus* aspiration studies that delivered as much as 2×10^6^ conidia [14], [15]. However, these results do support histopathological findings of previously reported murine intranasal [10] and whole body guinea pig studies [25]. Fogelmark and colleagues [25] demonstrated increased alveolar wall thickening with interstitial cells and granuloma formation following *A. fumigatus* exposure. Similarly, Murdock and colleagues observed goblet cell metaplasia following repeated exposures [10]. In the present study, inflammation appeared to be resolving by 48 and 72 hours, although, giant cells, and granulomas were still observed.

Microscopic evaluation of tissue sections demonstrated a small proportion of conidia (<10%) had become metabolically active (swollen conidia) by 4 hours and by 24–48 hours germ tubes had formed (<15% conidia). By 72 hours, conidia were nearly undetectable, likely processed and removed by neutrophils. Metabolically active conidia appeared to drive a mixed Th1/Th2/Th17 response that was characterized by the recruitment of neutrophils and eosinophils between 48–72 hours; however, these results should be interpreted with caution as the complete fungal lung burden was not assessed in the present study. These preliminary data suggest that as conidia began to swell and germinate, a unique T cell response appeared. BALF Th17 cells peaked at 48 hours in mice exposed to WT conidia that corresponded to the highest neutrophil counts in the BALF. Previous reports have shown that a major role for Th17 cells includes the recruitment of neutrophils, and this cell population has also been shown to contribute to viral persistence, allergy, chronic inflammation associated with parasitic infection and is important in immunity against opportunistic fungal pathogens, such as *Candida albicans* [27]–[29]. Recent intranasal and aspiration studies of *A. fumigatus* have also identified Th17 responses in the lungs of mice following repeated exposure [10],[14].

A CD8^+^ T cell response was additionally observed to correspond to *A. fumigatus* conidial germination. Compared to a previous study that showed the production of CD8^+^ IFNγ^+^ cell population following *A. fumigatus* aspiration [15], approximately 1/3 of the total CD8^+^ T cells were a CD8^+^ IL17^+^ (Tc17) cell population that peaked at 24 hours. The Tc17 population correlated with WT conidia germination, as well as the highest concentrations of mononuclear cells, neutrophils, and eosinophils present in the BALF. These results are consistent with the reported role for Tc17 cells in innate cell recruitment [30], [31]. CD8^+^ T cells have been shown to be protective by Carvalho and colleagues [32]; however, the role of Tc17 cells has remained less clear. Tc17 cells are a unique subset of CD8^+^ T cells associated with viral immunity (viral clearance), pulmonary inflammatory responses, systemic lupus erythematosus, control of tumor growth, and contact dermatitis [30]. Tc17 cells demonstrate functional plasticity and are reported to produce proinflammatory cytokines and chemokines responsible for the recruitment of neutrophils [31]. Recently, aspects of CD8^+^ and Tc17 immunity to *A. fumigatus* have been reported by our laboratory [14] as well as for the dimorphic fungal pathogens, *Blastomyces dermatitidis* and *Histoplasma capsulatum* [30]. Interestingly, the Tc17 response in this study was also specific for germinating conidia, as exposures to *Δalb1* conidia that did not germinate and polystyrene beads of the equivalent aerodynamic diameter did not induce the recruitment of Tc17 cell populations in the BALF (data not shown).

CD4 T^+^ cell responses within the MLNs of mice exposed to WT conidia increased over time, likely indicating continual antigen presentation, lymphocyte activation, and clonal expansion within this site [33], [34]. Levels of CD4^+^ IFN-γ^+^ (Th1), IL-13^+^ (allergy-associated Th2), as well as IL-17^+^ and IL-10^+^ (immunoregulatory) cells peaked at 72 hours in mice exposed to WT conidia and indicated a mixed Th1, Th2 and Th17 response. Compared to WT exposed mice, there were minimal differences in these cell numbers in mice exposed to *Δalb1* conidia, with the exception of IFN-γ production. This result was expected in the MLNs, as melanin is known to suppress proinflammatory responses [35]; however, MLN responses to *Δalb1* conidia have not been previously examined. We hypothesize that the low number of *Δalb1* conidia observed at 4 hours is below a threshold that would result in the proliferation of lymphocytic populations.

In this study, we additionally compared specific immunoglobulin responses in WT and *Δalb1* exposed mice. Serum titers of total IgE and sIgG were increased in WT compared to control and *Δalb1* exposed mice. In Western blots, no specific-IgM was detectable; however, 10 IgG-reactive proteins were observed. The WT exposed group shared similar immune reactivity with WT and *Δalb1* conidia and hyphal extracts. WT IgG reacted to several enzymes of broad intracellular function similar to those previously identified to be biomarker antigens associated with farmers lung [36]. These results further support the importance of fungal germination in expressing antigen/allergens following exposure to fungi [37]–[39]. In contrast, mice exposed to *Δalb1* conidia mounted an IgG response toward hydrophobin, a protein constituent of the cell wall of *A. fumigatus* conidia that was primarily identified as the major antigen in the proteomic analysis [40]. Although WT serum IgG also detected hydrophobin, the reactivity was weak compared to *Δalb1* exposed mice. In contrast to previously reported data by Aimanianda et al. [40] that showed hydrophobin to mask conidial recognition from the immune system, our findings suggest that the presence of melanin may block hydrophobin immune recognition. These findings support recent data presented by Chai et al. [35] that demonstrated fungal melanin may protect the conidia long enough to allow them to germinate. These data suggest that in the absence of melanin, conidia are recognized and killed more efficiently by the innate system. Although fungal germination would be restricted to thermotolerant organisms, these findings demonstrate that hyphal exoantigens are additional sources of antigen following repeated exposures. In order to determine the role of other sources of fungal exposure, such as hyphal and submicron fragments, additional characterization using this murine model will be required [41].

In conclusion, a novel inhalation exposure system was developed at NIOSH that allowed dry fungal conidia aerosols to be repeatedly delivered to mice housed in a nose only multi-animal exposure chamber. The generated aerosols reflect the potential burden of fungi encountered by humans in certain high exposure settings. The resultant pulmonary immune response from repeated inhalation exposures had a mixed Th1/Th2/Th17 cytokine profile. Tc17 cells were additionally identified in response to *A. fumigatus* germination. This T cell population may be an important source of IL-17 responsible for heightened recruitment of innate phagocytes observed at 24 and 48 hours following the final exposure. Additionally, we observed the production of specific IgG to prominent antigens derived from WT and *Δalb1* conidia and hyphal extracts. This study provides further insight into the role of conidial melanin in the induction of immune responses and demonstrates that sIgG could be produced to hydrophobin. Future studies that evaluate other mold species of environmental and occupational significance are required to provide further insight into the resulting pulmonary immune responses. Other potential uses of this inhalation model include studies of the pathogenesis of either acute or chronic aspergillosis and the influence of different immunosuppressive agents on the establishment of opportunistic fungal infections. These studies may identify additional biomarkers of fungal exposure that could prove useful in immunodiagnostics but more importantly, may provide further data to determine limits of personal and occupational exposure.

## Supporting Information

Data S1 **Supporting file containing original datasets associated with each manuscript and supporting information figure.**
(ZIP)

Figure S1 **Average stimulation indices (SI) determined by a modified local lymph node assay.** Mice were exposed to the indicated deposition concentration of WT conidia. SI were calculated as average disintegration per minute of the exposed group/average disintegration per minute control group. Horizontal line at SI of 10 is an arbitrary index used to determine positive lymphocyte proliferation in these studies. n = 10 mice/group.
(TIF)

Figure S2 **Histopathology of sections derived from** ***A. fumigatus Δalb1*** **exposed mice.** A) Representative histopathology sections from WT exposed mice sacrificed at the indicated time points. Top panel-H&E stained sections at 100× objective, Bottom panel-PAS stained sections at 10× objective.
(TIF)

Figure S3 **Flow cytometry analysis of intracellular cytokine production in the BALF. CD4^+^ and CD8^+^ T cells were quantified by multiplying the frequency of each individual cell population by the total cell counts.** Mice were sacrificed at the indicated time points after the 8th exposure. Data are presented as the average ± standard error of measure. n = 7–10 mice/group per time point. ****P≤0.0001, ***P≤0.001, *P≤0.05.
(TIF)

Figure S4 **Intracellular cytokine flow cytometry analysis of the MLNs. CD4^+^ and CD8^+^ T cells were quantified by multiplying the frequency of each individual cell population by the total cell counts.** Mice were sacrificed at the indicated time points after the 8th exposure. Data are presented as the average ± standard error of measure. n = 7–10 mice/group per time point. ****P≤0.0001, ***P≤0.001, *P≤0.05.
(TIF)

Figure S5 **Total IgE quantification via ELISA.** Sera from exposed mice was obtained at 3 days or 7 days following the final exposure, and analyzed via a commercially available total IgE ELISA kit (Biolegend, San Diego, CA). Each sample was prepared in duplicate and the values presented are the average ± standard error of measure.
(TIF)

Figure S6 **Specific IgG from** ***A. fumigatus*** **WT exposed mice.** Two dimensional Western blot analysis of specific IgG in serum from WT mice exposed to proteins generated from an *A. fumigatus* WT hyphal extract.
(TIF)

File S1 **Supporting file containing additional BALF and MLN results.**
(DOCX)
