# Supplementary figures and images for: A Murine Inhalation Model to Characterize Pulmonary Exposure to Dry Aspergillus fumigatus Conidia
Source: PLoS One. 2014 Oct 23;9(10):e109855. doi: 10.1371/journal.pone.0109855 (PMC4207673; doi:10.1371/journal.pone.0109855)

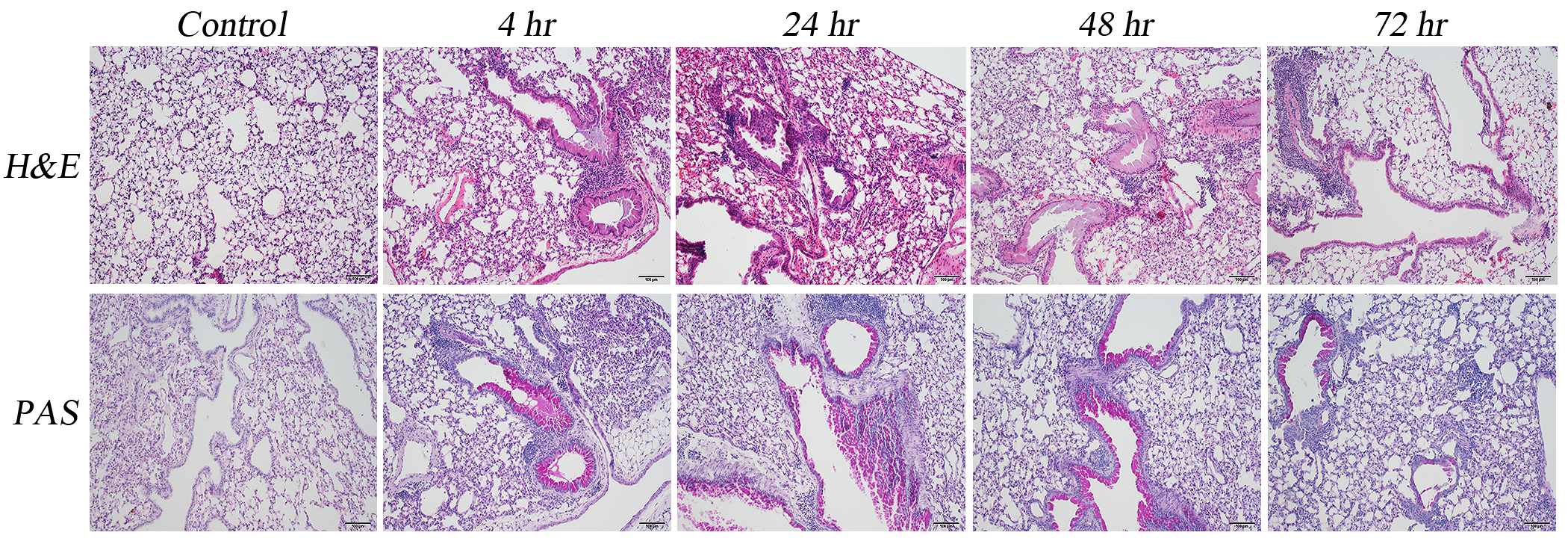

Supplement: Figure S2 — Histopathology of sections derived from A. fumigatus Δalb1 exposed mice. A) Representative histopathology sections from WT exposed mice sacrificed at the indicated time points. Top panel-H&E stained sections at 100× objective, Bottom panel-PAS stained sections at 10× objective. (TIF) [file pone.0109855.s003.tif]

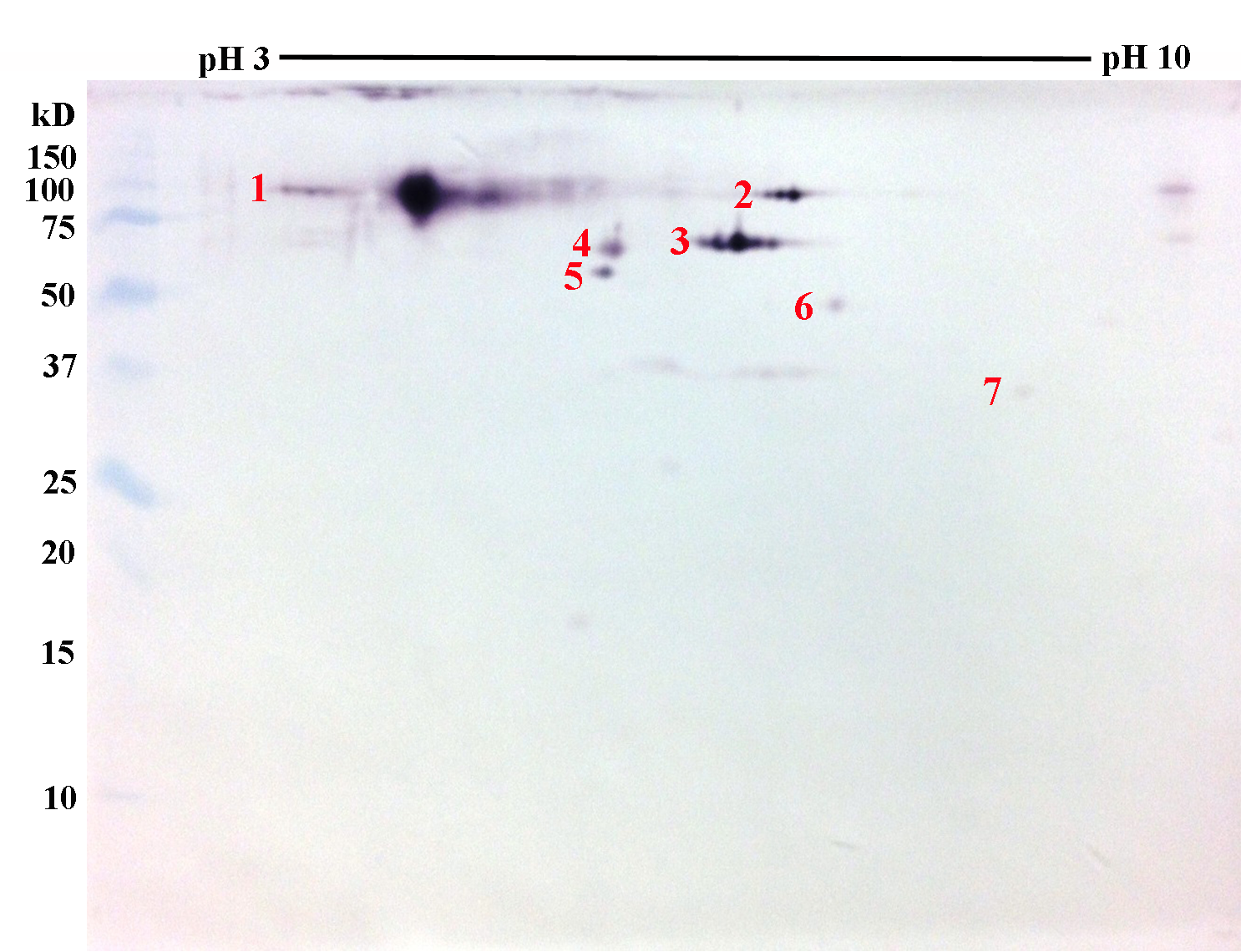

Supplement: Figure S6 — Specific IgG from A. fumigatus WT exposed mice. Two dimensional Western blot analysis of specific IgG in serum from WT mice exposed to proteins generated from an A. fumigatus WT hyphal extract. (TIF) [file pone.0109855.s007.tif]
