# Supplementary material for: A Murine Inhalation Model to Characterize Pulmonary Exposure to Dry Aspergillus fumigatus Conidia
Source: PLoS One. 2014 Oct 23;9(10):e109855. doi: 10.1371/journal.pone.0109855 (PMC4207673; doi:10.1371/journal.pone.0109855)
Supplement: File S1 — Supporting file containing additional BALF and MLN results. (DOCX) [file pone.0109855.s008.docx]

**A murine inhalation model to characterize pulmonary exposure to dry *Aspergillus fumigatus* conidia**

Amanda D. Buskirk^1^, Brett J. Green^1^, Angela R. Lemons^1^, Ajay P. Nayak^1^, W. Travis Goldsmith^2^, Michael L. Kashon^3^, Stacey E. Anderson^1^, Justin M. Hettick^1^, Steven P. Templeton^1^, Dori R. Germolec^4^, Donald H. Beezhold^1*^

1. Allergy and Clinical Immunology Branch, Health Effects Laboratory Division, National Institute for Occupational Safety and Health, Centers for Disease Control and Prevention, Morgantown, WV 26505, USA.
2. Pathology and Physiology Research Branch, Health Effects Laboratory Division, National Institute for Occupational Safety and Health, Centers for Disease Control and Prevention, Morgantown, WV 26505, USA.
3. Biostatistics and Epidemiology Branch, Health Effects Laboratory Division, National Institute for Occupational Safety and Health, Centers for Disease Control and Prevention, Morgantown, WV 26505, USA.
4. Toxicology Branch, DNTP/NIEHS, Research Triangle Park, NC 27709

*Corresponding author

Donald H. Beezhold, PhD FAAAAI

Allergy and Clinical Immunology Branch

National Institute for Occupational Safety and Health

Centers for Disease Control and Prevention

1095 Willowdale Road

Morgantown, WV 26505

Email: [zec1@cdc.gov](mailto:zec1@cdc.gov)

**Supplemental Results**

In BALF, Th1 (CD4^+^IFN-γ^+^) and Th2 (CD4^+^IL-13^+^) T cells were significantly higher at 24 and 72 hour time points in mice exposed to *Δalb1* conidia. Similar to the responses observed for each cytokine, CD4^+^TNFα^+^ and CD4^+^IL-10^+^ populations were higher at 4 and 48 hours than at 24 and 72 hours in mice exposed to WT conidia (Figure S3). Aside from the CD4^+^IL-17^+^ T cell population identified in this study, the remaining CD4 T cell populations did not appear to be associated with conidial germination (Figure S3).

Immediately following exposure, there was a mixed CD8 T cell response in BALF as shown by the significantly increased levels of CD8^+^IFN-γ^+^, elevated CD8+ TNFα^+^ and CD8^+^IL-13^+^ T cells at 4 hours (Figure S3). CD8^+^IL-10^+^ T cell numbers increased as levels of CD8^+^IFN-γ^+^ and CD8^+^ TNFα^+^ declined (Figure S3). These results additionally corresponded to the kinetics of conidia germination (Figure 2C). These data suggest that Tc17 (CD8^+^ IL-17A^+^) cells may respond to and have a function role in the presence of germinating *A. fumigatus* conidia. Compared to repeated control aspiration exposures to polystyrene beads, these results were specific for *A. fumigatus* and were not the result of innate particulate clearance or overburden (data not shown).

In the MLN analysis, Th1 Th2 populations were also significantly elevated in the *Δalb1* exposure groups at 4 hours; however, this mixed response was delayed in the WT exposure group and observed at 24 hours (Figure S4).
